# Effect of Herbal *Echinacea* on Recurrent Minor Oral Aphthous Ulcer

**DOI:** 10.2174/1874210601812010567

**Published:** 2018-08-31

**Authors:** Faezeh Khozeimeh, Zahra Saberi, Atefeh Tavangar, Fahime Fakhari Badi

**Affiliations:** 1 Dental Research Center, Department of Oral and Maxillofacial Medicine, School of Dentistry, University Of Medical Sciences, Isfahan, Iran; 2 Dental Material Research Center, Department of Oral and Maxillofacial Medicine, School of Dentistry, Isfahan University Of Medical Sciences, Isfahan, Iran; 3 School of Dentistry, Isfahan University Of Medical Sciences, Isfahan, Iran

**Keywords:** Minor oral aphthous, Herbal medicines, *Echinacea*, Oral mucosa problem, Herpetiformis ulcers, Water-soluble polysaccharides

## Abstract

**Background:**

The oral aphthous is a common oral ulcer with intense pain and there is no treatment for it, yet. *Echinacea* is an herbal medicine that moderated the immune system.

**Objective:**

The aim of this study was to investigate the effects of *Echinacea* on the treatment of aphthous ulcer.

**Methods:**

50 patients with minor aphthous participated in our study. 25 patients take 3 tablets in a day for five weeks (case group) and 25 patients didn’t take any tablets (control group). The patients were monitored for one month before taking the tablets for six months. During this period, the number of lesions, complete improvement of ulcers, recurrence rate and intensity of pain were considered in each month. Finally, the Friedman and ANOVA tests used to analyze the obtained data.

**Result:**

Our study showed a significant difference between a number of lesions during six-month in case and control groups (*p*>0.001). In this way, we observed that the number of lesions was decreased significantly after six months in the case group. Hence, ANOVA analysis showed a significant decrease between each month for the intensity of pain (*p*=0.025), complete improvement (*p*<0.001) and recurrence rate (*p*=0.026).

**Conclusion:**

In conclusion, we showed that *Echinacea* tablets as an herbal medicine have positive effects on a number of lesions, intensity of pain, complete improvement and recurrence rate in patients with recurrent minor aphthous ulcers.

## 1 INTRODUCTION

An aphthous ulcer is a common oral mucosa problem, which is a solitary lesion, circular, symmetric and shallow [1]. The clinical symptoms of aphthous have three categories: minor aphthous ulcers, major aphthous ulcers and herpetiformis ulcers. Etiology of aphthous is unclear and treatment methods are non-specific and based on experimental studies [2]. Aphthous treatments should be proportionate to its intensity and symptoms. Nowadays, many medicinal plants are used for the treatment of aphthous, such as mouthwash of Salvia (*Salivia oficinalis*), chamomile, Carrot, pomegranate (*Punica granatum*), Water Melon (*Cantaloupe*), wild geranium, Aloe vera and liquorice [3]. Some plant compounds have been proposed as reinforcing the immune system in the treatment of diseases involving the immune system, which can be noted to the *Echinacea purpurea* plant [4]. From the early 17^th^ century, the natives of America used *Echinacea* to treat snake bites, gums and mouth diseases, colds, cough, sore throat and blood poisoning. Moreover, this plant used to treat scarlet fever, syphilis, malaria and diphtheria, traditionally. The *Echinacea* effects in improvement of ulcer and disinfection were discussed and approved in 1920 [5]. The chemical components of *Echinacea* plant species include lipophilic, water-soluble polysaccharides, caffeic acid derivatives and chicoric acid [6]. The alkaloids, polysaccharides and chicoric acid in this plant were stimulating the immune system [7]. The polysaccharides in this plant have anti-inflammatory properties and stimulate the fibroblasts to repair damaged connective tissue [8]. The polysaccharide in this plant stimulates the immune system by increasing the production of T cells and other white blood cells, activation of macrophages and monocytes, and increase production of polymorphonuclear (PMN) [8, 9]. Each *Echinacea* tablet contains 114 mg dried leachate of *Echinacea purpurea* plant, which does not recommend its oral administration for more than eight weeks and non-food consumption more than three weeks [5, 8].

Since recurrent aphthous is one of the most common oral diseases, a large percentage of people suffer from this disease. A significant cause of aphthous was impaired immune system, therefore the aim of this study was to investigate the efficacy of *Echinacea* tablets on the complications of recurrent minor oral aphthous ulcer disease.

## 2 MATERIALS AND METHODS

This study is a clinical trial. 50 patients were selected among those referred persons to the centers and dental clinics in Isfahan-Iran with minor aphthous ulcers. The subjects were diagnosed with minor aphthous over the past year, which occurs at least once in two months. The pregnant and lactating patients, consumers of systemic steroids, immune system modulators and non-steroidal anti-inflammatory drugs, as well as patients with a history of ulcerative colitis, Crohn disease, Behcet's disease, and severe anemia, life-threatening diseases such as severe heart, kidney and liver disease were excluded from this study.

After completing the form of informed consent, 25 patients received 105 *Echinacea* tablet as case group (Immustim Tablet, Flower medicine pharmacy, Iran). The oral administration of the drug program was started from first day of the next recurrent aphthous for five weeks, and 3 tablets daily. Thus, patients from a recurrent before drug use to recurrences occurred after taking the drug were monitored monthly, in six months. The number of lesions, duration of complete improvement (according to day), recurrent intervals and pain intensity (according to VAS) were recorded at each recurrence. Also, the other 25 patients don’t use any tablet within six months (control group).

Finally, obtained results within 6 months were analyzed using the *SPSS* software (version 22). The Friedman test and analysis of variance were used for repeated data analysis (α=0.05). This clinical trials code is (IRCT) IRCT2016012626209N1.

## 3 RESULTS

All subjects were monitored from a recurrent before taking the drug to recurrent after taking the first drug monthly, for six months. Each month, the average number of lesions, pain intensity, duration of complete improvement and recurrent intervals were calculated and finally, the periods in case and control groups were compared.

**Fig. (1):**
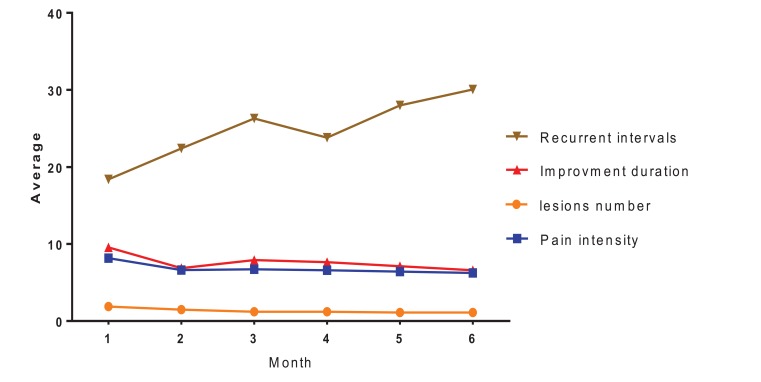
Compare of the variables average in the six-month period. The obtained results showed significant differences between numbers of lesions, average of pain intensity, duration of complete remission and recurrent intervals in one-month intervals.

The obtained results from the Friedman test showed significant differences between numbers of lesions in one-month intervals and between case and control groups. Also, variance analysis (ANOVA) for frequent data showed significant differences between the average of pain intensity, duration of complete remission and recurrent intervals in a period of six months, and between case and control groups (Table **1** and Fig. **1**). Finally, paired sample t-test and Wilcoxon tests have been shown a significant difference between the studied variables (Table **2**).

## 4 DISCUSSION

In this study, the effect of *Echinacea* tablets was studied on aphthous ulcers. Based on obtained results in this study, this drug has a positive effect on the number of lesions, intensity of pain, duration of complete improvement and recurrent intervals in patients. These effects may be due to the presence of polysaccharides in the *Echinacea* plant and stimulate the immune system. Thus, this plant is used for the treatment of common cold, respiratory tract infection, and also viruses ulcers such as syphilis, abscesses ulcers and swelling of the tonsils [4, 8, 9].

*Echinacea* is most widely used to strengthen the immune system against viral diseases that weaken the immune system. Therefore, topical administration of *Echinacea* preparations has positive effects in accelerating the improvement of ulcers and tissue repair. It is believed that this effect is related to inhibiting the activity of tissue hyaluronidase enzyme by substances contained in this plant [10].

In a study by Pascal P and Viguier S (2000), the efficacy of a daily dose of prednisolone (1mg/kg) and colchicine (2 mg/d) in 5 patients with aphthous was investigated. They demonstrated that prednisolone leads to the improvement of ulcers quickly and colchicine decreased the pain [11]. In the present study, we showed that the *Echinacea* has similar effects with prednisolone (accelerate to improvement) and colchicine (reduction of pain) on patients with aphthous. *Echinacea* tablet is an herbal medicine and has fewer side effects than prednisone and colchicine, use of the *Echinacea* seems more appropriate in treating aphthous.

A study by Ilia Volkou *et al*., (2009) showed that the use of vitamin B12 by patients with aphthous leads to reduce pain, number of lesions and duration of improvement [12]. In this regard, Matlabnejad *et al*., (2008) investigated the effect of tea plant extraction (*Hypericum perforatum*) on 30 patients with recurrent oral aphthous ulcers. They observed that 5.0% of *Hypericum perforatum* in mouthwashes has a positive impact on pain and improvement of minor aphthous ulcers [13]. In the present study, in addition to reducing of pain and accelerating the improvement of recurrent aphthous, the *Echinacea* plant leads to decrease the number of aphthous ulcers and increase the recurrent intervals, which indicates the significant influence of this drug on the aphthous disease.

A study by Jahanshahi and Saniei (2012) examined the effect of triamcinolone drug on patients with aphthous disease. They showed that this drug in the second session decreases the size of the lesions (*P* = 0.026) and pain intensity (*P*=0.043) [14]. In the present study, in addition to reduce pain, reduce the number of lesions and recurrent, and accelerate improvement. This study showed that *Echinacea* reduces the size of lesions in some patients. Of course, the aim of this study was not to investigate the size of lesions and recommend it to be considered in future research. Generally, according to this study, we can introduce the *Echinacea* as a first systemic drug for the treatment of aphthous ulcers.

The limitations of this study can refer to patient cooperation; because systemic drug consumption and lack of other drugs consumption in six months are difficult for patients. In this study, the effects of *Echinacea* drug were not compared with other drugs. Therefore, it is recommended that comparison must be done between this drug and other effective drugs on aphthous, in future studies.

## CONCLUSION

The *Echinacea* tablet as a herbal medicine provides beneficial effects on the number of aphthous ulcers, intensity of pain, duration of complete improvement and recurrence intervals in patients with recurrent minor oral aphthous ulcer; and can be considered as an effective and affordable treatment method.

## Figures and Tables

**Table 1:** Mean and standard deviation indicator of improvement, within six months.

**Ave/SD**	**First Month**	**Second Month**	**Third Month**	**Fourth Month**	**Fifth Month**	**Sixth Month**
Number of Lesions	(0**±**0.97) 1.88	(0**±**0.54) 1.48	(0**±**0.38) 1.21	(**0±**0.4) 1.22	(0**±**0.28) 1.10	(0±0.033) 1.12
Pain Intensity	(0**±**1.44) 8.17	(1±0.77) 6.63	(2**±**0.17) 6.71	(2**±**0.1) 6.59	(2**±**0.9) 6.42	(0±2.19) 6.24
Remission Duration	(3**±**0.33) 9.57	(3**±**0.33) 6.88	(3**±**0.47) 7.91	(3**±**0.26) 7.65	(3±7.35) 7.12	(0±6.60) 6.60
Recurrent Intervals	(14**±**0.35) 18.39	(12**±**0.23) 22.4	(17**±**0.56) 26.3	(15**±**0.6) 23.81	(19±0.55) 28.0	(19±0.01) 30.06

SD: Standard Deviation; Ave: Average.

**Table 2:** Compare of *p*-value between the number of lesions, intensity of pain and improvement duration.

**Recurrent Intervals**	**Remission Duration**	**Pain Intensity**	**Number of Lesions**	**Month**
0.000	0.003	0.000	0.012	1 and 2
0.002	0.033	0.001	0.003	1 and 3
0.004	0.004	0.004	0.002	1 and 4
0.001	0.001	0.000	0.000	1 and 5
0.000	0.000	0.000	0.000	1 and 6
0.263	0.553	0.927	0.034	2 and 3
0.168	0.160	0.957	0.033	2 and 4
0.227	0.964	0.429	0.002	2 and 5
0.018	0.211	0.254	0.010	2 and 6
0.972	0.933	0.742	0.465	3 and 4
0.931	0.082	0.4.6	0.201	3 and 5
0.011	0.003	0.279	0.223	3 and 6
0.613	0.006	0.023	0.129	4 and 5
0.008	0.000	0.010	0.102	4 and 6
0.115	0.003	0.391	0.656	5 and 6
**ANOVA:** PV=0.026	**ANOVA:** PV<0.001	**ANOVA:** PV=0.025	**Friedman:** PV<0.001	**General Test**

ANOVA: Variance Analysis
